# Hyperhomocysteinemia: An Unusual Cause of Budd-Chiari Syndrome

**DOI:** 10.7759/cureus.94033

**Published:** 2025-10-07

**Authors:** Sai Samhitha Mogalapu, Swathika T M, Keerthana P, Namicharan Nabirajan, Roshan Raj, Sahasyaa Adalarasan

**Affiliations:** 1 Institute of Internal Medicine, Madras Medical College, Chennai, IND

**Keywords:** budd-chiari syndrome, caudate lobe hypertrophy, hepatic vein thrombosis, hyperhomocysteinemia, portal vein thrombosis

## Abstract

Budd-Chiari syndrome (BCS) is a rare disorder caused by hepatic venous outflow obstruction, with clinical presentation ranging from asymptomatic disease to acute liver failure. We report a case of secondary BCS due to hyperhomocysteinemia in a 52-year-old male patient with a history of alcohol use. He presented with jaundice, vomiting, and melena. Examination showed icterus and tender hepatomegaly, while laboratory investigations revealed elevated liver enzymes. Doppler ultrasound and contrast-enhanced CT of the abdomen demonstrated caudate lobe hypertrophy, intrahepatic IVC and portal vein thrombosis, and non-visualization of hepatic veins, consistent with BCS. A hypercoagulable workup revealed elevated homocysteine levels, with other parameters within the normal range, confirming hyperhomocysteinemia as the underlying etiology. The patient was managed with anticoagulation, initially with heparin followed by warfarin. Hyperhomocysteinemia, though rare, should be considered in the evaluation of BCS, as prompt diagnosis using imaging and laboratory studies allows timely intervention and improved outcomes.

## Introduction

Budd-Chiari syndrome (BCS) has an incidence of 1 in 100000, making it a rare disorder [1]. It is caused by occlusion of the hepatic veins draining directly into the inferior vena cava. The obstruction of the venous architecture leads to venous hypertension. The most common lobe involved is the caudate due to its direct drainage into the inferior vena cava (IVC) [2]. The clinical features develop after the occlusion of two hepatic veins, which causes an increased interstitial fluid and liver congestion, resulting in hypoxic damage to the liver [3]. This results in the classic triad of jaundice, ascites, and tender hepatomegaly. The course can be acute or chronic and can be associated with portal hypertension. Portal vein thrombosis is observed in thrombophilic disorders [4]. The etiology is generally attributed to the myeloproliferative neoplasms, malignancy, infections, use of oral contraceptives, and hypercoagulable states secondary to inherited mutations in protein C, protein S, and factor V, antithrombin 3, which account for 80% of the cases; 20%, however, are idiopathic, and the cause is unidentified [2]. This is a case of hyperhomocysteinemia causing BCS, a rare often unidentified etiology.

## Case presentation

A 52-year-old man presented with features of vomiting for four weeks associated with food particles and blood streaks in the vomitus, yellowish discoloration of the eyes for 10 days, and dark colored urine for 10 days with melena for two days. He is a known alcoholic, consumes about three drinks per week, and has been a smoker for over 20 years. On examination, the patient was icteric. Vitals were normal with a pulse rate of 82/ min, BP of 115/80mm Hg, afebrile status, and O2 saturation at 98% in room air. On examination, tender hepatomegaly with the liver edge felt 7 cm below the right costal margin moves with respiration, tender, and firm in consistency. There was no free fluid in the abdomen. Bowel sounds were present.

Investigations revealed microcytic hypochromic anemia with reduced platelet count (Table 1). Liver function tests showed elevated direct and indirect bilirubin (Table 2).

**Table 1:** Blood investigations done on the patient MCV: Mean cell volume; MCH: Mean corpuscular hemoglobin; MCHC: Mean corpuscular hemoglobin concentration

Lab Parameters	Values	Reference Range
Total leucocyte count	8900 cells/mm^3^	4000 - 11000
Red blood Cells	3.4 million/ml	4.5 - 6.5
Hemoglobin	8.5 g/dl	Dec-16
Hematocrit	32	40 - 46
Platelets	140,000/ml	150,000 - 350,000
MCV	75.6 fL	79 - 101
MCH	23 pg/cell	27 - 33
MCHC	30	32 - 36

**Table 2:** Liver enzyme values on days 1, 3, 5, and 6 AST: Aspartate aminotransferase; ALT: Alanine aminotransferase; ALP: Alkaline phosphatase; TB: Total bilirubin; DB: Direct bilirubin

Day	1	3	5	6	Reference Range
AST /ALT (IU/L)	96/23	65/18	68/16	99/19	5-40/7-56
ALP (IU/L)	241	180	163	199	44-147
TB/DB (mg/dL)	9.6/4.2	12.5/11.0	10.0/7.1	13.2/11.6	0.3-1.2/0-0.3
Total protein (g/dL)	7.6	7.3	7.1	7.1	6-8.3
Serum albumin (g/dL)	3.1	2.9	2.3	3.0	3.5-5

**Figure 1:**
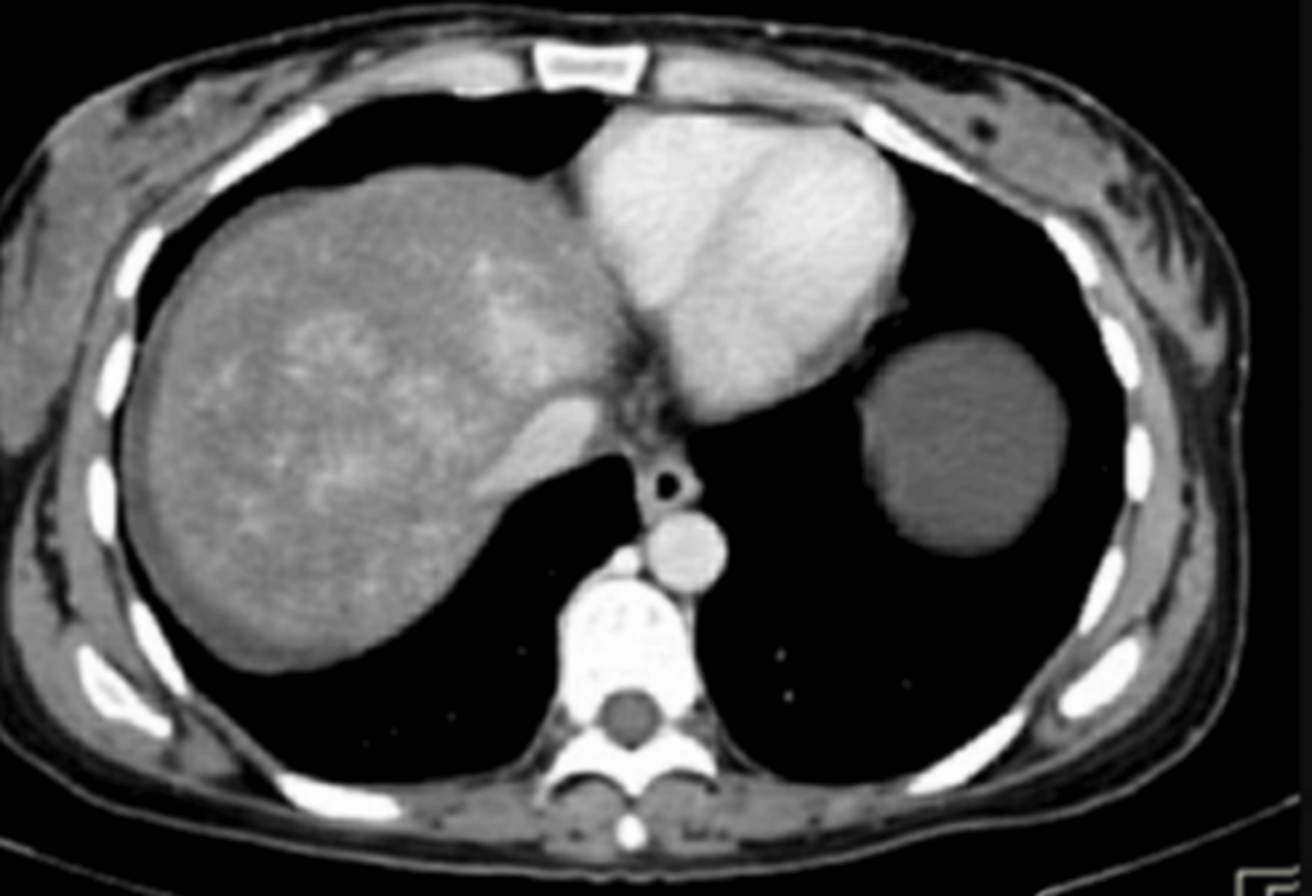
Triple-phase CT of abdomen showing non-visualization of the hepatic veins in the portal venous phase

**Figure 2:**
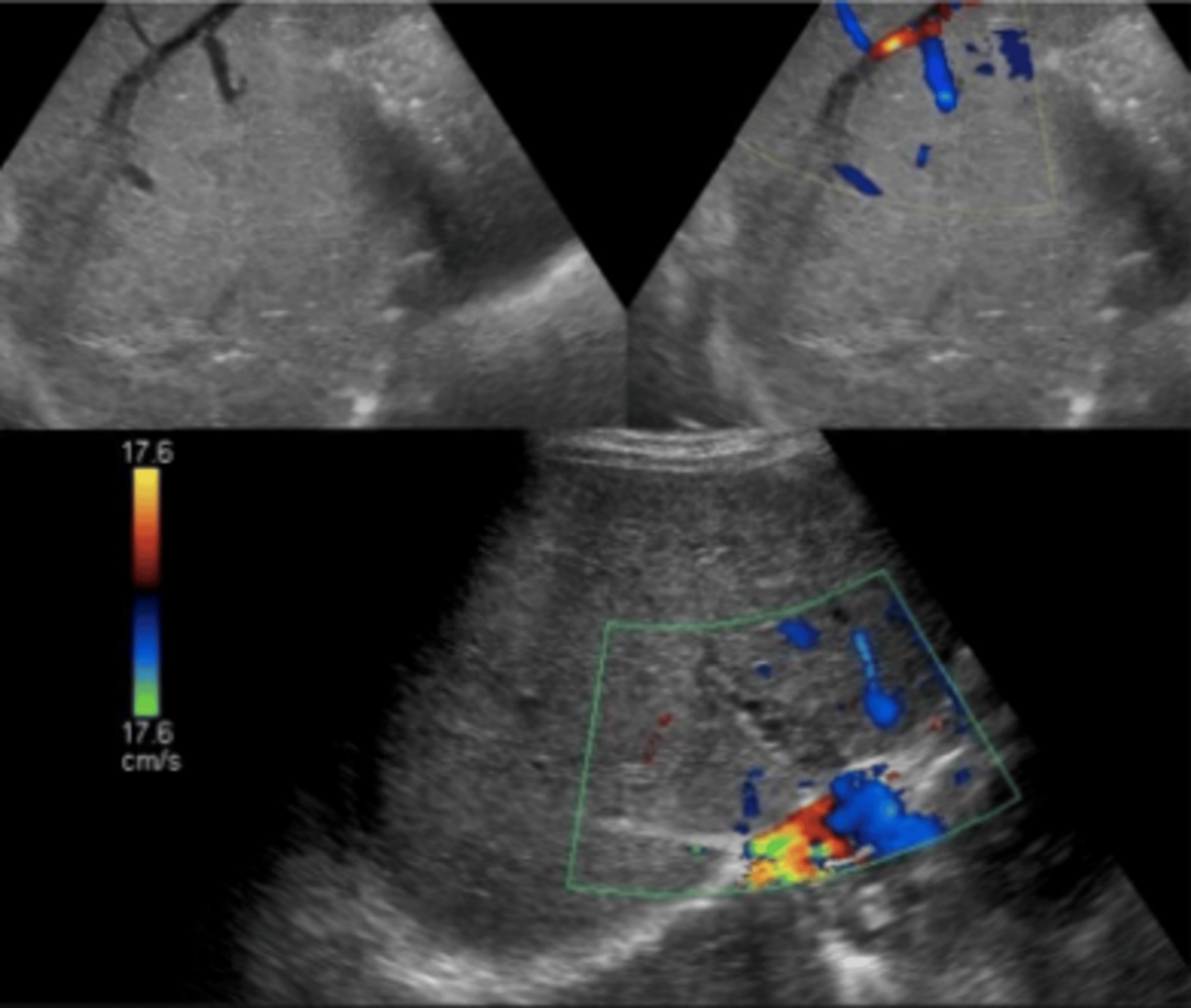
Ultrasound portal venous Doppler shows no color flow within the right and middle hepatic veins with a patent left hepatic vein. Turbulent flow noted within the intrahepatic IVC probably due to intrahepatic IVC thrombosis with features suggestive of Budd-Chiari syndrome IVC: Inferior vena cava

Stool occult blood was positive with negative viral markers, stool ova cyst negative, and normal serum AFP. Ultrasound abdomen with portal venous Doppler revealed grade 1 fatty liver with splenomegaly, hepatomegaly with coarse echos and caudate lobe hypertrophy; the intrahepatic branches of the portal vein showed evidence of thrombosis with the development of multiple intrahepatic collaterals (Figure 1). The CECT abdomen triple phase showed thrombosis at the intrahepatic portion of the IVC, with thrombosis of the portal vein, superior mesenteric vein, with caudate lobe hypertrophy (Figure 2).

Esophagogastroduodenoscopy revealed the presence of portal hypertensive gastropathy. A diagnosis of BCS was hence made after ruling out other causes. The patient was further worked up for hypercoagulability. Serum homocysteine was found to be elevated. Serum vitamin B12 and methylmalonic acid levels were normal. 

The patient was started on prophylactic antibiotics (1 g/day i.v. ceftriaxone for seven days). The patient was also started on 5000 IU (given twice daily) of heparin intravenously for seven days. The patient was instructed accordingly and discharged from the hospital.

## Discussion

The etiology and presentation of BCS are varied [5]. The etiology for the same is quite varied and unpredictable; some rare causes that have been reported include COVID-19, polycythemia, pregnancy, etc. The presentation of BCS is different in adults and children. Primary BCS is most commonly seen in children, and secondary BCS is seen in adults. The most common presentation in children includes jaundice with hepatomegaly [6], while ascites occurs in adults [7]. The causes of secondary BCS include a malignant infiltration of the vein, compression of the vein by abscess, cyst, APLA, and the inflammation following this can also cause a procoagulant state, which leads to thrombosis. The causes of primary BCS are most commonly due to inherited thrombophilias [5,7]. The common causes of BCS secondary to thrombosis of the portal vein include protein S, protein C, and antithrombin 3 deficiency [5].

The investigation paved the way to narrow down the diagnosis, as the ultrasound investigation revealed a grade 1 fatty liver with splenomegaly with otherwise normal liver contour. To further investigate the presence of liver secondaries and gastrointestinal malignancies, a CECT abdomen was done, which revealed the thrombosis of the intrahepatic portion of the inferior vena cava with superior mesenteric vein thrombosis, along with caudate lobe hypertrophy. The diagnosis of Budd-Chiari syndrome was made, but the etiology had to be delineated. The absence of a mass obstructing the venous drainage was ruled out in the CECT. Based on the features in the imaging, one could come to the conclusion that the duration of the illness is subacute in nature due to the caudate lobe hypertrophy, presence of nodularity of the liver, non-visualization of the hepatic veins, and the development of intrahepatic collaterals [8]. The nodularity of the liver is attributed to the hemorrhagic necrosis of the parenchyma and compensatory volume expansion.

Furthermore, the presence of thrombosis gave direction towards the elevated procoagulant factors. The levels of ANA, beta 2 glycoprotein, anti-cardiolipin antibody (IgG/IgM), protein C, protein S, antithrombin 3, Factor V leiden, and serum homocysteine levels were evaluated. The values were normal for factors except serum homocysteine, which was elevated. In a study conducted to analyze the cause of BCS among West and China, the most common cause among the thrombophilic etiology was hyperhomocysteinemia, followed by cardiolipin antibodies [9]. High homocysteine levels are associated with an increased prothrombotic action because they enhance platelet activation, increase thrombin generation, argument factor V, and impair fibrinolytic activity. This is also associated with vascular injury and endothelial dysfunction [10].

The treatment of this disease can be surgical or medical management, depending on the etiology. The surgical options include shunting using mesocaval or mesoatrial shunts, orthotopic liver transplantation, and radiological shunting. The outcome of the patients varied depending on the treatment. In this case, the coagulation pathway dysfunction was hence managed by heparin 5000 IU intravenous and later bridged with warfarin from day 3 with a target INR range of 2-3. Serial INR was monitored weekly until stable values were reached, and then monthly INR monitoring was done.

## Conclusions

BCS can present across all age groups with variable manifestations and carries a significant risk of mortality. It should therefore be considered as a differential diagnosis in patients presenting with abdominal pain, ascites, jaundice, or hepatomegaly. Early recognition and identification of the underlying cause of hypercoagulability are crucial for improving patient outcomes. In this case, timely diagnosis enabled effective management and a favorable clinical course.

Hyperhomocysteinemia, though uncommon, is an important and potentially overlooked etiology of secondary BCS. Incorporating homocysteine level assessment into the hypercoagulable workup is recommended. The patient discussed in the present case study highlights that prompt diagnosis through laboratory investigations and imaging modalities not only facilitates appropriate management but also significantly improves prognosis.

## References

[1] Valla DC (2003). The diagnosis and management of the Budd-Chiari syndrome: consensus and controversies. Hepatology.

[2] Hitawala AA, Gupta V: (2025). Budd-Chiari syndrome. StatPearls [Internet].

[3] Akiyoshi H, Terada T (1999). Centrilobular and perisinusoidal fibrosis in experimental congestive liver in the rat. J Hepatol.

[4] Gonzalez-Flecha B, Reides C, Cutrin JC, Llesuy SF, Boveris A (1993). Oxidative stress produced by suprahepatic occlusion and reperfusion. Hepatology.

[5] Egesel T, Büyükasik Y, Dündar SV, Gürgey A, Kirazli S, Bayraktar Y (2000). The role of natural anticoagulant deficiencies and factor V Leiden in the development of idiopathic portal vein thrombosis. J Clin Gastroenterol.

[6] Zhou WJ, Cui YF, Zu MH, Zhang QQ, Xu H (2016). Budd-Chiari syndrome in young Chinese: clinical characteristics, etiology and outcome of recanalization from a single center. Cardiovasc Intervent Radiol.

[7] Samanta A, Sen Sarma M, Yadav R (2023). Budd-Chiari syndrome in children: challenges and outcome. World J Hepatol.

[8] Bansal V, Gupta P, Sinha S (2018). Budd-Chiari syndrome: imaging review. Br J Radiol.

[9] Undas A, Brozek J, Szczeklik A (2005). Homocysteine and thrombosis: from basic science to clinical evidence. Thromb Haemost.

[10] Slakey DP, Klein AS, Venbrux AC, Cameron JL (2001). Budd-Chiari syndrome: current management options. Ann Surg.

